# Polychlorinated biphenyls in mussels, small pelagic fish, tuna, turtles, and dolphins from the Croatian Adriatic Sea waters: an overview of the last two decades of monitoring

**DOI:** 10.2478/aiht-2024-75-3814

**Published:** 2024-03-29

**Authors:** Snježana Herceg Romanić, Gordana Mendaš, Sanja Fingler, Vlasta Drevenkar, Bosiljka Mustać, Gordana Jovanović

**Affiliations:** Institute for Medical Research and Occupational Health, Zagreb, Croatia; University of Zadar, Department of Ecology, Agronomy and Aquaculture, Zadar, Croatia; University of Belgrade Institute of Physics, Belgrade, Serbia; Singidunum University, Belgrade, Serbia

**Keywords:** artificial intelligence, environmental pollution, explainable artificial intelligence, marine environment, Mediterranean Sea, PCBs, persistent organic pollutants, POPs, Jadransko more, morski okoliš, objašnjiva umjetna inteligencija, onečišćenje okoliša, PCB, POP, postojana organska zagađivala, Sredozemno more

## Abstract

This review summarises our two decades of polychlorinated biphenyl (PCB) monitoring in different marine organisms along the eastern Adriatic Sea. The aim was to gain an insight into the trends of PCB distribution in order to evaluate the effectiveness of past and current legislation and suggest further action. Here we mainly focus on PCB levels in wild and farmed Mediterranean mussels, wild and farmed bluefin tuna, loggerhead sea turtles, common bottlenose dolphins, and small pelagic fish. The use of artificial intelligence and advanced statistics enabled an insight into the influence of various variables on the uptake of PCBs in the investigated organisms as well as into their mutual dependence. Our findings suggest that PCBs in small pelagic fish and mussels reflect global pollution and that high levels in dolphins and wild tuna tissues raise particular concern, as they confirm their biomagnification up the food chain. Therefore, the ongoing PCB monitoring should focus on predatory species in particular to help us better understand PCB contamination in marine ecosystems in our efforts to protect the environment and human health.

## Abstract (hr)

U ovom preglednom članku sažeto se opisuju rezultati našega monitoringa polikloriranih bifenila (PCB) u različitim morskim organizmima u istočnom Jadranskom moru u protekla dva desetljeća. Cilj je bio steći uvid u trendove raspodjele PCB-a kako bi se procijenila učinkovitost prošlih i postojećih zakonskih propisa te predložilo daljnje djelovanje. Fokus je na važnosti Jadranskoga mora, razinama PCB-a u divljim i uzgojenim mediteranskim školjkama, divljoj i uzgojenoj plavoperajnoj tuni, glavatim kornjačama, običnim dobrim dupinima i maloj plavoj ribi. Metodama umjetne inteligencije i napredne statistike omogućen je uvid u utjecaj različitih varijabli na unos PCB-a u istraživane organizme kao i njihovu međusobnu ovisnost. Naši rezultati pokazuju da su razine PCB-a u malim plavim ribama i školjkama odraz globalnoga onečišćenja okoliša, a visoke razine u tkivima dupina i divlje tune posebno su zabrinjavajuće. Potreban je daljnji nadzor PCB-a u morskom okolišu, s naglaskom na predatorske vrste.

Polychlorinated biphenyls (PCBs) are a group of 209 synthetic, aromatic, and organic compounds (known as congeners) commercially produced as mixtures with different chlorine content, mostly known as Aroclor (trade name by Monsanto Corporation, the major US producer from 1930 to 1977), Clophen (Germany), Fenclor (Italy), Kanechlor (Japan), and Phenoclor (France). PCB are a very persistent and have remained widespread in the environment, including the Arctic and Antartica in the 21^st^ century, even though their production and usage have been banned or limited in numerous industrial countries since the 1970s and 1980s (1).

PCBs meet specific criteria to be considered persistent organic pollutants (POPs) (2), one of which is long-range transport in the atmosphere. There they vary between the gaseous and particulate phase, which determines their fate. PCB constantly travel between air and earth surfaces (e.g., vegetation, soil, surfaces of rivers, lakes, oceans, and seas), and this dynamic re-cycling involves constant exchange between environmental compartments (air and water surface, air and soil, plants, water and soil or sediment). They are also subject to long-range transport via air masses in gaseous or particulate phase and can penetrate deep into the soil, oceans, and sediments, where they persist.

Such persistence in the environment poses pollution risks that remain to this day and stem from the past uses in electrical equipment and construction materials, now deposited in landfills or kept at industrial facilities, from which they leach into soil and water. The issue is further aggravated by illegal dumping of PCB-containing materials, accidental spills in transport or handling, new primary sources, particularly from materials exported to Asia and Africa (3), certain industries, and forest fires (1).

PCBs reach the marine environment through atmospheric deposition, rivers, other surface waters, sediments, and wastewaters discharged directly into the sea. They mainly bind to particulate matter in the water column and accumulate in sediments. On the global level, the oceans are the final sink/destination of persistent compounds and consequently act as secondary sources of contamination due to slow PCB decomposition. They subsequently enter the marine food chain via lower trophic-level organisms, gradually making their way to predators. Khairy et al. (4) reported PCBs in mammals from Antarctica, and Jepson et al. (5) have found that in marine mammals they reach the highest levels, exceeding the threshold of 9 mg/kg lipid for the onset of physiological effects in experimental mammal studies. In some cetaceans such as killer whales and bottlenose dolphins in the northeast Atlantic and in many cetacean species of the Mediterranean Sea these levels reach above 50 mg/kg lipid and exceed the highest PCB toxicity threshold of 41 mg/kg lipid for marine mammals (6). Globally, killer whales are the most highly PCB-contaminated species on Earth (5). These findings may in part explain the long-term decline in European whale populations from 1990 to 2012 (5).

Much scientific attention has been given to the Mediterranean marine environment because of its cultural, economic, ecological, and geographic importance. A considerable increase in pollution through rivers, atmospheric depositions, and discharges from the coast has been degrading the marine ecosystems throughout the last few decades. Another concern is the accumulation of POPs owed to a limited exchange of water mass with the Atlantic Ocean. Monitoring programmes established to assess the current state and to identify potential sources seek to find better ways of pollution control through national and international regulatory limits, such as the Stockholm Convention on Persistent Organic Pollutants (7) and EU legislation (8).

The aim of this review is to take a closer look at our own research (9,10,11,12,13,14,15,16,17,18,19,20) carried out since 2000 at the Institute for Medical Research and Occupational Health in Zagreb, Croatia, as our studies make up the bulk of all PCB research in marine organisms in the Croatian part of the Adriatic Sea, which covers almost the entire eastern Adriatic coast. Research conducted before 2000 has been summarised in a comprehensive review article by Picer et al. (21).

To put our research in a broader context, we searched related literature available on the Web of Science, Scopus, and Google Scholar using the following keywords: “persistent organic pollutants,” “polychlorinated biphenyls,” “marine environment,” and “Adriatic Sea”. Additionally, we studied the research conducted by Milun et al. (22,23,24) on PCBs in Mediterranean mussels and bivalves, as well as research conducted within the MYTIAD project, which was focused on assessing chemical contamination of Adriatic coastal waters through current mussel biomonitoring (25).

## THE ADRIATIC SEA

The Adriatic Sea is a semi-enclosed basin of the Mediterranean that extends over 138,000 km^2^. Although the Adriatic is known as an oligotrophic sea, its shallow coastal waters are characterised by higher nutrient input from rivers, lower salinity, and greater zooplankton and pelagic fish spawn area than open waters (26). It is home to a diverse array of marine organisms, ranging from microscopic plankton to large apex predators.

According to the Croatian Bureau of Statistics for 2020, of the 68,232 t of fish caught in the Croatian part of the Adriatic, more than 95 % were pelagic [e.g., sardine (*Sardina pilchardus*); anchovy (*Engraulisen crasicolus*), and bluefin tuna (*Thunnus thynnus*)], of which 77.1 %, were sardines. In 2020, the mariculture production of 18,275 t was dominated by seabream (*Sparus aurata*, 7,792 t), seabass (*Dicentrarchus labrax* 6,754 t), and bluefin tuna (2,611 t). Furthermore, Croatia, like other Mediterranean countries, strives for species diversification in aquaculture and has recently introduced meagre (*Argyrosomus regius*) and common dentex (*Dentex dentex*) into the Adriatic Sea farms (27).

Although relatively shallow, the Adriatic is known for its biodiversity thanks to a variety of seabed sediments, such as rocky, sandy, and muddy. However, the last few decades have seen changes in its flora and fauna owed to the climate change (especially the rise in temperature and higher salinity), anthropogenic activity, and Lessepsian migration. It faces problems with new allochthonous species that are mostly thermophilic and have expanded their habitats migrating northward. Some of the new species come from the ballast water or aquaculture and are potentially invasive. Overall, the number of Adriatic fish species had grown from 407 in 1996 to 456 in 2017 (28). Since the Croatian part of the Adriatic covers more than 35 % of the total Croatian territory, monitoring changes in marine ecosystems and the conservation of its biodiversity are of great importance (29).

## DETERMINATION AND INTERPRETATION OF PCB CONCENTRATIONS IN ADRIATIC MARINE ORGANISMS

Of the 209 listed PCB congeners, our research has primarily been focused on the six so-called “indicator PCBs” (PCB-28, PCB-52, PCB-101, PCB-138, PCB-153, PCB-180), because they are commonly used as indicators of overall PCB contamination in a given environment. These congeners are often found in commercial PCB mixtures and are prevalent in environmental samples. By monitoring the levels of these indicator PCB, scientists and environmental researchers can gain insight into the extent of PCB pollution and potential risks to ecosystems and human health.

We have also studied 11 toxicologically relevant congeners (PCB-60, PCB-74, PCB-105, PCB-114, PCB-118, PCB-123, PCB-156, PCB-157, PCB-167, PCB-170, and PCB-189). These congeners are distinguished from indicator congeners, insofar as they are known to have greater toxic or bioaccumulation potential and are of greater concern in risk assessment and regulatory contexts. They are often analysed separately to better understand their impact and to develop appropriate management strategies.

The concentrations of either indicator or toxicologically relevant PCBs in various marine organisms are often given either for each individual congener or for their sums (∑Ind PCB and ∑ToxRel PCB, respectively).

In our research (9,10,11,12,13,14,15,16,17,18,19,20) all samples were analysed at the Biochemistry and Organic Analytical Chemistry Unit of the Institute for Medical Research and Occupational Health, Zagreb, Croatia. Statistical analysis and modelling with artificial intelligence (AI) algorithms through machine learning were done in collaboration with the University of Belgrade Institute of Physics, Belgrade, Serbia (13,14,15, 17,18,19,20).

Filter feeding organisms, such as mussels, have been proven effective as indicator organisms in environmental monitoring programmes worldwide, owing to their abundance and capacity to accumulate a wide spectrum of contaminants. In order to establish baseline concentrations of PCBs, we investigated wild and farmed Mediterranean mussel (*Mytilus galloprovincialis*) sampled in the coastal waters of the eastern Adriatic. Wild mussel populations were collected at 14 locations along the Croatian coast in March 2006 (10), and our findings showed that ΣInd PCB levels were consistently higher than the ΣToxRel PCB levels (Table 1). Among indicator PCBs, PCB-138 and PCB-153 dominated with concentrations at least twice as high as any of the rest, followed by PCB-52, PCB-28, PCB-101, and PCB-180. The most dominant ∑ToxRel PCBs were PCB-118, PCB-60, PCB-170, and PCB-114. Spatial PCB distributions were relatively consistent throughout, with particularly high levels observed near areas of intense industrial activity such as large shipyards, yacht marinas, industrial zones, and urban centres. These areas emerged as hot spots for PCB pollution, indicating local sources of contamination. One sampling site located in a major port and industrial hub (Rijeka Bay) recorded the highest PCB levels on the Croatian coast. An earlier monitoring study of this location conducted by Picer and Picer (30) covering the period from 1972 to 1992 concluded that PCB levels remained unchanged over those 20 years and that their sources were atmospheric deposition, local urban and industrial wastewater, and maritime activities.

**Table 1:** A summary of PCB levels in tissues of different species from the eastern Adriatic (Croatia) reported over the last 20 years

**Species (year of sampling, *n*)**	**Concentrations (ng/g of wet mass unless otherwise indicated)**			**Reference**
	**ΣInd PCBs (median and range)**	**ΣToxRel PCBs (median and range)**	**ΣPCBs (median and range)**	
**Mussels (*Mytilus galloprovincialis*)**				
**Wild (2006, 14)**	27.39 (7.13–74.43)^a^	9.7 (3.44–37.72)^a^	35.98 (10.6–112.2)^a^	(9)
**Farmed (2010, 32)**				(10)
Winter (January)	1.4 (0.88–12.27)	0.53 (0.25–1.91)	1.88 (1.12–14.11)	
Summer (July)	3.99 (2.22–17.54)	1.49 (0.55–7.38)	5.87 (3.23–23.86)	
**Mussels (*Mytilus galloprovincialis*, *Ostrea edulis*, *Venus verrucosa*, *Arca noae*, *Callista chione*)**				(20)
**Farmed and wild (2012, 13)**				
Autumn (November)	3.56 (1.43–14.35)^a^			
Spring (May)	4.21 (1.29–19.67)^a^			
**Small pelagic fish (2014–2016, 105**)				(18)
Horse mackerel (*Trachurus trachurus*)	0.70 (0.15–3.06)	0.32 (0.11–1.04)		
Sardine (*Sardina pilchardus*)	2.96 (0.43–9.63)	1.30 (0.26–5.54)		
Round sardinella (*Sardinella aurita*)	0.57 (0.31–1.3)	0.26 (0.16–0.47)		
Chub mackerel (*Scomber japonicus*)	0.80 (0.1–1.69)	0.39 (0.09–0.69)		
Anchovy (*Engraulisen crasicolus*)	0.38 (0.2–2.35)	0.26 (0.12–0.92)		
**Bluefin tuna (*Thunnus thynnus*)**				
**Wild (1996, 9)**				(15)
White muscle	838 (413–1184)^b^	378 (132–616)^b^	1155 (545–1800)^b^	
**Farmed (2015, 7)**				(14)
Liver (n=6)	31.40 (15.85–38.84)	10.03 (4.33–11.32)	41.43 (20.18–50.16) 80.3 (78.4–116.0)^b^	
Muscle (n=7)	19.39 (10.54–36.75)	6.33 (3.62–8.81)	24.43 (14.16–45.50) 148.14 (93.7–234.9^b^	
Branchiae (n=7)	6.66 (3.99–10.02)	2.93 (1.51–3.92)	9.34 (6.45–13.69)	
**Loggerhead sea turtles *(Caretta caretta*) (2001–2002, 26)**				(8)
	260 (126–2587)^c^		312 (177–2934)^c^	
	538 (184–4036)^b^		655 (274–4577)^b^	
**Common bottlenose dolphins *(Tursiops truncatus*) (2000–2005, 13)**				(11)
Blubber (n=13)	10899 (391–51619)		16904 (704–71913) 49 (2–494)^d^	
Liver (n=10)	271 (63–1544)		480 (167–2504) 74 (4–676)^d^	
Kidney (n=7)	204 (41–1825)		365 (63–2621) 209 (29–3744)^d^	
Lung (n=7)	232 (57–688)		391 (193–964) 92 (37–1383)^d^	
Muscle (n=12)	240 (22–6621)		413 (42–9031) 122 (20–557)^d^	
Heart (n=8)	78 (58–1634)		253 (110–2303) 305 (9–2661)^d^	

n=number of samples.

a in ng/g dry mass;

b in ng/g lipid mass;

c in ng/g fat tissue wet mass;

d in mg/kg lipid mass

Continuing our investigation, we aimed to compare the results obtained from the analysis of both wild and farmed Mediterranean mussels. The farmed mussels were collected at 15 shellfish breeding farms situated along the central and southern Croatian Adriatic coast in 2010. Our findings (11) were similar to those observed in wild mussel populations (10), with ∑Ind PCB exceeding ∑ToxRel PCB at all sampling locations (Table 1). The dominant compounds were PCB-138 and PCB-153, followed by PCB-52, PCB-28, PCB-101, and PCB-180. The most prominent congeners in the ToxRel PCB group were PCB-123 and PCB-170. The prevalence of hexachlorinated congeners PCB-153 and PCB-138 in mussels aligns with reports for other Mediterranean locations (31,32,33).

We also observed a significant seasonal variation in contaminant concentrations in farmed mussels; higher levels were recorded in the summer, and lower levels in the winter. However, the contaminant levels remained within tolerable daily intake limits of 2 pg/kg per day (34). Like us, Milun et al. (22) observed seasonal variations in PCB concentrations in wild and cultivated bivalve molluscs *(Mytilus galloprovincialis, Ostrea edulis, Venus verrucosa, Arca noae*, and *Callista chione*) collected at 11 wild and two mariculture sites along the Croatian coast in May and November 2012. They also observed variations between sampling sites and suggested potential pollution sources, such as historical industrial activities, long-term urban and industrial wastewater discharge, and the proximity to a nautical marina and local shipyard. In another study, Milun et al. (23) found a significant increase in PCB concentrations, most notably PCB-138 and PCB-153, in wild Mediterranean mussel from Kaštela Bay, known for its steel and cement plant, brewery, food and beverage production, port, shipyard, and a former PVC chlor-alkali facility.

Mussels were also monitored for PCB contamination within the frame of the MYTIAD project focused on the accumulation of PCB-28, PCB-31, PCB-52, PCB-101, PCB-105, PCB-118, PCB-138, PCB-153, PCB-156, and PCB-180 (25). High PCB levels were reported for Taranto, Italy, with concentrations reaching 114.4 µg/kg dry mass, followed by Baošići and Kotor in Montenegro, where PCB levels slightly exceeded 60 µg/kg dry mass.

The research conducted between 2014 and 2016 on sardines, anchovy, round sardinella (*Sardinella aurita*), chub mackerel (*Scomber japonicus*), and horse mackerel (*Trachurus trachurus*) caught along the eastern Adriatic (19) revealed the prevalence of indicator congeners PCB-153, PCB-138, and PCB-180, whereas toxicologically relevant PCB-118 and PCB-170 levels were consistent with previous reports for the Adriatic (35) and Ionian fish species (36). Vuković et al. (14) observed the following pattern of accumulation: anchovy<chub mackerel<horse mackerel<sardines. To assess the health risks for consumers of small pelagic fish, we adopted the Risk Assessment Information System models to address specific local conditions and ran a comprehensive benefit-risk analysis based on essential fatty acid content in these fish (20). The richest sources of omega-3 fatty acids were anchovy and round sardinella, while chub mackerel and sardine followed closely. Sardines had the highest levels of docosahexaenoic acid and eicosapentaenoic acid, both essential for human health. It was reassuring to find out that PCB levels in these fish species did not pose a significant health risk to consumers, and the benefit-risk ratios were consistently below 1, which means that the consumption of these fish varieties has more benefits than potential risks for human health.

Our research extended further to predatory species to gain insights into the distribution of PCBs across the trophic levels of the Adriatic Sea and to understand how the levels measured in predatory species may impact human health. To this end we analysed PCBs in the muscle (because it is consumed as food), liver (because it can tell us a lot about PCB metabolism), and gills (as the entry point of organic contaminants from water) of bluefin tuna farmed in the central Croatian Adriatic in January 2015 (15). Indicator PCB-153, PCB-138, PCB-153, and PCB-180 dominated in all tissues, whereas PCB-118, PCB-123, and PCB-170 dominated among the toxicologically relevant PCBs in tuna liver and muscle, but not in the gills. The concentrations (ng/g lipid mass) of ∑Ind PCB and ∑ToxRel PCB followed the muscle>liver>gill sequence, correlating with the percentage of tissue lipids. Vizzini et al. (37) reported a similar pattern of higher muscle than liver contamination in tuna farmed in Sicily, Italy, although the reported concentrations of Σ43 PCBs (which includes the indicator and toxicologically relevant ones) were significantly higher than our findings (371.74 ng/g per lipid mass in the liver and 1916.98 ng/g in the muscle). Furthermore, the highest concentrations of ΣInd PCBs we found in farmed tuna muscles (15) and small pelagic fish (19) was lower than the maximum permissible level of 75 ng/g wet mass in fish set by the European Commission (38).

The Mediterranean Sea pollution with PCBs raises particular concern for endangered species, such as wild tunas, sea turtles, and dolphins. PCB levels determined in the muscle tissue of wild bluefin tuna sampled in the Adriatic in 1996 were among the highest reported in the literature (Table 1), raising concern about bioaccumulation through the food web (16). Similarly, Chiesa et al. (39) suggest that the Mediterranean Sea could be more polluted than the Pacific, Indian, and Atlantic Ocean, judging by the highest PCB levels found in the Mediterranean tuna.

Speaking of endangered species, one Adriatic study (9) addressed the anthropogenic impact on loggerhead turtles (*Caretta caretta*) by measuring PCBs in their fat tissue and provided an additional insight into PCB bioaccumulation along the food web. These measurements were made in 26 turtles found dead between June 2001 and November 2002. PCB-153 median concentrations per lipid mass (244.6 ng/g) were the highest, followed by PCB-138 (169.1 ng/g), PCB-180 (50.4 ng/g), and PCB-118 (33.4 ng/g). These levels are higher than reported for loggerheads from the Italian part of the Adriatic (40) but lower than reported for Greece and Cyprus (41).

We also investigated PCB contamination of the common bottlenose dolphin (*Tursiops truncatus*) in 13 specimens found stranded at different locations along the northeast coast of the Adriatic Sea between 2000 and 2005 (12). Their PCB profile in all tissues (Table 1) was also dominated by three indicator congeners, PCB-153, PCB-138, and PCB-52, in that order. An analysis of PCBs in the blubber samples of delphinids from the south-eastern Brazilian coast (42) suggests that low-chlorinated PCBs, such as PCB-52 found in our study, travel over long distances as they bind to suspended particulate matter in the atmosphere. In our study (12), PCB congeners were distributed similarly across the analysed dolphin tissues: hexachloro>pentachloro>heptachloro>tetrachloro> trichlorobiphenyls, and their levels decreased in the following order: blubber>muscle>kidney>liver>heart>lung. Furthermore, their lipid-normalised concentrations were significantly higher than in the wild or farmed tuna and loggerhead sea turtles (Table 1).

Our findings are similar to those reported by Genov et al. (43) in common bottlenose dolphins found in the Gulf of Trieste and the adjacent waters in the northern Adriatic between 2011 and 2017, but both are much higher than those reported for dolphins found along the Adriatic coast in Italy (7.25–56.96 µg/g lipid mass for Σ17 PCBs) (44) or those found along the western Mediterranean coast in 2000–2003 (45, 46). According to Storelli and Marcotrigiano (44), sum PCB levels in blubber exceeding 50 µg/g wet mass might present a health risk to cetaceans, yet in our study, only two blubber samples exceeded this threshold.

As for PCB-138 and PCB-153, they resist metabolism up the food web, are regularly found at the highest levels in marine organisms (46,47,48,49), and are suitable for comparison within and between species (50). In this context, our PCB-153 results suggest that the endangered species of wild tuna, loggerhead sea turtles, and dolphins from the eastern Adriatic Sea are among the most affected by PCB pollution worldwide.

### Modelling insights

Considering how complex the datasets of environmental PCB monitoring in marine organisms can be, new modelling methods have been developed recently to complement traditional statistical analysis. These new modelling methods seek to explain combined effects of a number of environmental variables that influence the fate of pollutants in marine environments, such as temporary or permanent direct and indirect emission sources, pollutant interactions, their metabolic behaviour, their dependence on meteorological conditions, and the hierarchy in the food chain. The usual methods such as factor analysis, principle component analysis, and latent class analysis fail to “capture” pollutant interactions and their non-linear interrelations and dependencies (51). However, the development of artificial intelligence with algorithms based on machine learning have already brought significant improvements to disciplines such as social science and economics, and could bring even greater benefits to environmental sciences (52, 53). To manage the complexity, heterogeneity, and non-linearity of environmental data, these algorithms allow for sophisticated data processing, which in combination with the so called *explainable artificial intelligence*, could improve on human intuition and discover seemingly hidden associations between variables and predictors without the explicit knowledge of underlying processes (54).

In our preliminary modelling study (13), we applied advanced classification and clustering methods – self-organising maps (aka Kohonen maps, SOM) and decision tree (DT) learning – to explore species- and season-specific POP dependencies in *Cyprinidae* fish sampled in Vransko Lake. Vransko Lake is situated in a shallow karst bed and separated from the Adriatic by a narrow karst ridge. Seawater enters the lake through the karst and vice versa, which greatly influences seasonal variations in water levels and salinity and helps to better understand processes in the Adriatic Sea.

SOM and DT combined indicated that the season and meteorological parameters, including temperature, atmospheric pressure, and relative air humidity, affected the uptake of almost all POPs in the tissue of the investigated species. The exceptions were PCB-138 and PCB-153, whose uptakes were generally even across seasons.

Another study (14) showed that the distribution of PCB-52, PCB-60, PCB-74, PCB-101, PCB-105, PCB-118, PCB-156, PCB-157, PCB-167, PCB-170, and PCB-180 in horse mackerel, chub mackerel, anchovies, and sardines varied between coastal and open sea waters and across sampling years. To investigate the underlying reasons for these differences, we employed a machine learning model known as eXtreme Gradient Boosting (XGBoost) in conjunction with the SHapley Additive exPlanations (SHAP). SHAP aids in explaining the output of machine learning models, allowing us to asses the influence of individual PCB levels, saturated fatty acids (SFAs), monounsaturated fatty acids (MUFAs), and polyunsaturated fatty acids (PUFAs) on the distribution of the indicator congener PCB-138 in these fish. Our analysis proved that PCB-138 is the most prevalent congener in fish besides PCB-153 but is more representative of PCB behaviour in fish. It is worth noting that SHAP and XGBoost are recognised for providing consistently accurate and locally relevant solutions (55), as validated in previous environmental studies (56). A “locally relevant solution” would be the one that accurately accounts for the unique environmental properties of a specific locality and provides insights or predictions that are pertinent to that specific area. The findings obtained with this model indicated a substantial impact of highly halogenated and consequently more persistent congeners, specifically PCB-153, PCB-118, PCB-180, and PCB-170, on the distribution of PCB-138.

This model also singled out two saturated (myristic and margaric) and two omega-3 and 6 (eicosadienoic and dihomo-γ-linolenic) acids as the most important for PCB-138 accumulation in sardine, anchovy, and mackerel. However, the nutritionally beneficial eicosapentaenoic and docosahexaenoic acid had no such impact on the uptake of organic pollutants. The two methods successfully explained the relationships between POPs and fatty acids and helped us to better understand pollutant behaviour than common statistical methods would have.

## CONCLUSION AND SUGGESTIONS FOR FURTHER RESEARCH

Our research of PCB levels in marine organisms from the Croatian Adriatic conducted over the last two decades has shown the dominance of PCB-138, PCB-153, PCB-180, and PCB-170. An interesting finding is that the last congener is more dominant in mussels (both wild and farmed) and farmed tuna than in other species. Small pelagic fish, in turn, revealed seasonal variations and site-specific differences in PCB levels.

Furthermore, our research has shown higher PCB accumulation in wild mussels and tuna than their farmed counterparts, in which the sum of indicator PCB did not exceed the permissible EU limit. This may be owed to controlled feeding and growth conditions.

Even though PCB levels in the Adriatic reflect global pollution, high levels in dolphins and wild tuna are particularly worrying, as they confirm their biomagnification along the food chain and higher pollution in the Mediterranean and Adriatic Sea than the world’s oceans. This is why our future research should focus on PCB exposure and toxicity in the Adriatic predatory species. We recommend establishing routine monitoring programmes specifically targeting predatory species known to bioaccumulate PCBs. These programmes should involve regular sampling of tissues such as muscle, liver, and adipose tissue to assess PCB levels and track trends in PCB contamination over time. This can provide valuable data on the effectiveness of regulatory measures and identify emerging contamination hotspots.

We also recommend informing consumers about healthy seafood choices and providing guidance on safe consumption practices.
